# Effect of the reaction medium on the characteristics of silanized titanium dioxide particles: Differences obtained in the Zeta potential data and infrared spectra

**DOI:** 10.1016/j.dib.2018.10.107

**Published:** 2018-10-26

**Authors:** E. Delgado Alvarado, L. López-Zamora, Cristina Pérez Pérez, E. Pérez, J.A. Vazquez-Lopez, J.A. González-Calderón

**Affiliations:** a Doctorado Institucional en Ingeniería y Ciencias de Materiales, Universidad Autónoma de San Luis Potosí, Zona Universitaria, Av. Dr Manuel Nava s/n, Lomas, C.P. 78290 San Luis Potosí, SLP, Mexico; b División de Estudios de Posgrado e Investigación. Tecnológico Nacional de México/Instituto Tecnológico de Orizaba, Av. Oriente 9 No. 852. Col. Emiliano Zapata, C.P. 94320 Orizaba, Veracruz, Mexico; c Departamento de Ingeniería Bioquímica, Instituto Tecnológico de Celaya, Av. Tecnológico y Antonio García Cubas s/n, C.P. 38010 Celaya, Gto, Mexico; d Instituto de Física, Universidad Autónoma de San Luis Potosí, Zona Universitaria, Av. Dr. Manuel Nava s/n, Lomas, C.P. 78290 San Luis Potosí, SLP, Mexico; e Departamento de Ingeniería Industrial, Instituto Tecnológico de Celaya, Av. Tecnológico y Antonio García Cubas s/n, C.P. 38010 Celaya, Gto, Mexico

## Abstract

In this document we present the differences in the Zeta potential and in the Infrared spectra data obtained from the characterization of silanized titanium dioxide particles, using two different solvents as reaction media: ethanol and toluene. Also, we provide micrographs of transmission electron microscopy in order to show morphological differences between the analyzed samples.

**Specifications table**

**Table:** 

Subject area	*Chemical Engineering*
More specific subject area	Colloid and Surface Chemistry
Type of data	*Transmission Electron Microscope images, Fourier Transformed Infrared Figure and Zeta Potential Figure.*
How data was acquired	**Transmission electron microscopy (TEM)**
	The images of the nanoparticles were obtained with a transmission electron microscope JEOL TEM-1010 operating at 100 kV.
	**Fourier Transform Infrared Spectroscopy (FTIR)**
	The curves of spectra were performed with the help of a Perkin-Elmer FTIR-ATR spectrometer, Model Spectrum 100.
	**Zeta Potential**
	The Zeta potential tests were performed with the help of a Delsa Nano C particle analyzer A53878 equipment. Solutions of NaOH and HCl at a concentration of 0.001% w/V were used to adjust the pH value using disposable cells equipped with two electrodes in solution for measurements.
Data format	*Analyzed*
Experimental factors	TiO_2_ nanoparticles were modified superficially by two different silanization processes. The coupling agent: 3-aminopropyl-triethoxy-silane (APTES) was added at a ratio 5: 1of (TiO_2_/APTES).
	For the incorporation of TiO_2_ in ethanol (Ethanol S-TiO_2_) [1] and in toluene (Toluene S-TiO_2_) [2], we followed the method described in previous reported works.
Experimental features	**Transmission electron microscopy (TEM)**
	The nanoparticles were suspended in distilled water and 5 μL of the dilution were placed on copper grids coated with carbon.
	**Fourier Transform Infrared Spectroscopy (FTIR)**
	The analysis included a range of wave number from 4000 to 1000 cm^−^^1^ with 60 scans, stacking the nanoparticles for a better reading at 25 °C.
	**Zeta Potential**
	The stability of the TiO_2_ particles and the silanized particles (S-TiO_2_) were evaluated in water through their precipitation time, driven by the gravity force at different pH in a range of 2–12, using a light source dual 30 mV and a 658 nm Laser Diode at 25 °C.
Data source location	San Luis Potosí, Mexico. [22.145297,-101.0183967]
Data accessibility	Mendeley Data, v1 http://dx.doi.org/10.17632/jdg6h7csd3.1
Related research article	L. López-Zamora, H.N. Martínez-Martínez, J.A. González-Calderón, Improvement of the colloidal stability of titanium dioxide particles in water through silicon based coupling agent, Mater. Chem. Phys. 217 (2018) 285–290. doi:10.1016/j.matchemphys.2018.06.063.

**Value of the data**

• The data are valuable to avoid further characterization of silanized particles under different reaction media.
• The data provide the detail information of Infrared Spectra patterns and Zeta Potential of silanized titanium dioxide particles using ethanol and toluene as reaction media.
• The data show the differences on the characteristics of silanized particles by effect of the reaction media used.
• Data was collected using the main solvents reported in the literature to silanized nanoparticles.

## 1 Data

In this document, we present the data obtained from the characterization of silanized titanium dioxide particles (TiO_2_) with 3-aminopropyl-triethoxy-silane (APTES)in two different reaction media: ethanol [1] and toluene [2]. The amount of silane added to the particles to be reacted was the same for the two experiments, and only the reaction media was changed. In the provided data we can see that there are significant changes in the physical, morphological and chemical characteristics of the silanized particles.

**Fig. 1:**
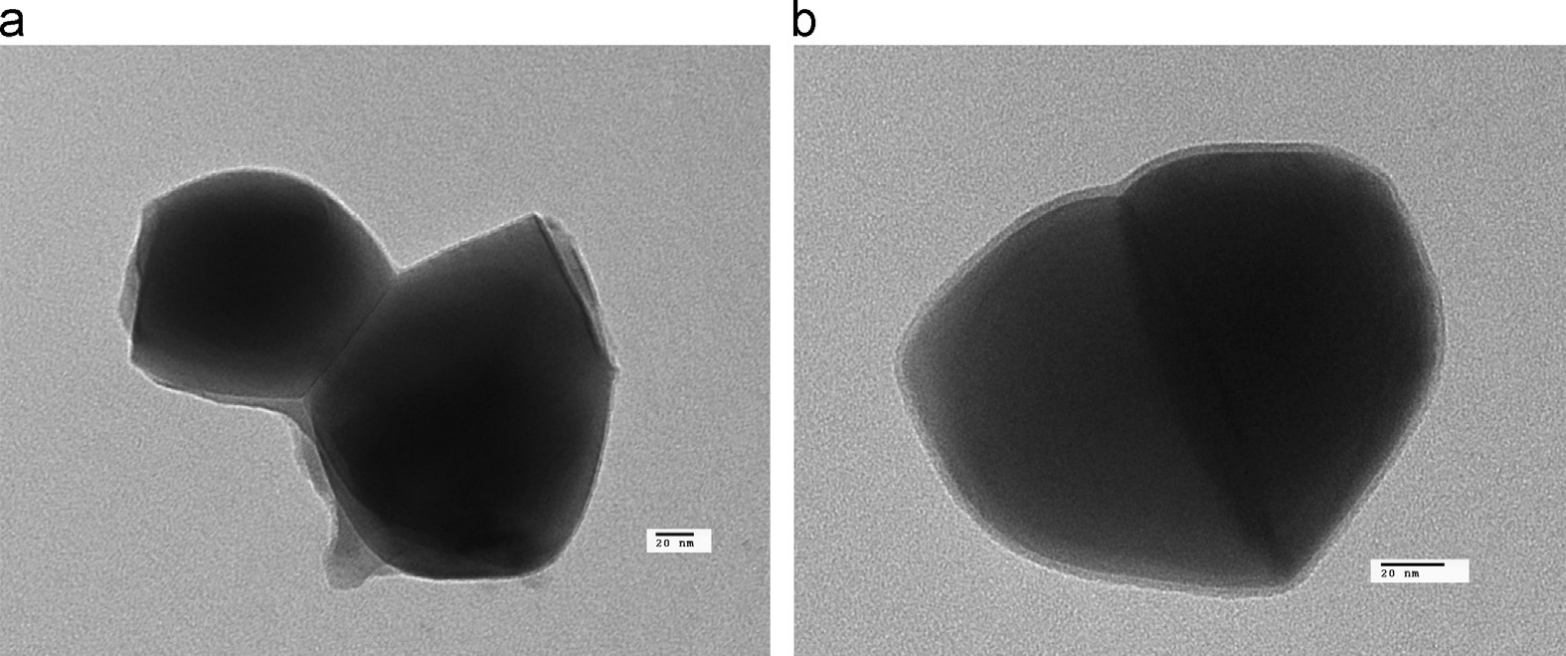
TEM images of the studied samples. (a) Toluene as reaction media and (b) Ethanol as reaction media.

In Fig. 1, we present the Transmission electron microscopy (TEM) images of the silanized samples, it can be seen that the toluene S-TiO_2_coating is thicker than the ethanol S-TiO_2_ particles; It shows that the silanization in toluene favors the encapsulation of the particles and a controlled reaction is carried out on the surface of each particle.

**Fig. 2:**
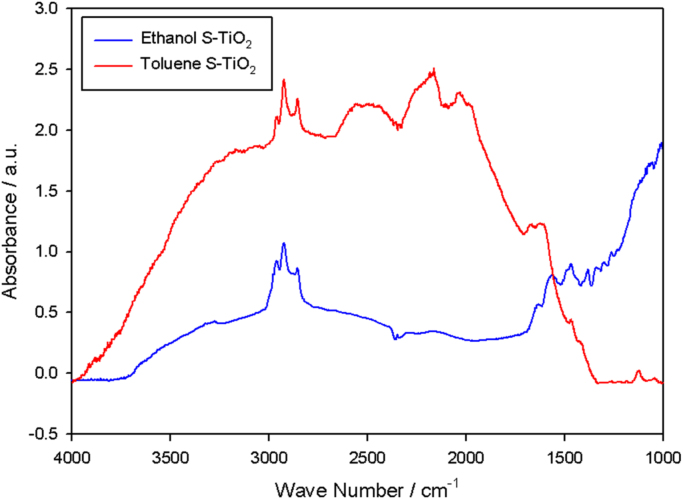
FTIR spectra of the silanized particles synthesized in different solvents as reaction media.

**Fig. 3:**
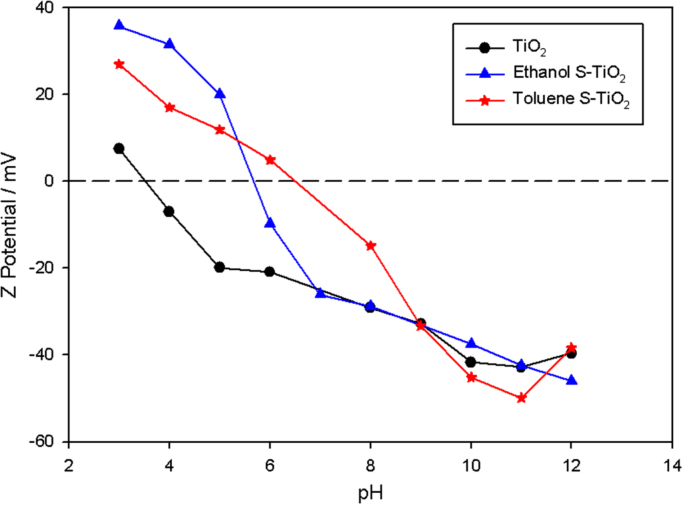
Zeta potential behavior of the studied particles.

In the Fig. 2, the FTIR spectra of the silanized particles show differences in the obtained bands; especially, the band attributed to the vibration of Si-O closes to 1000. Finally, in the Z Potential analysis presented in Fig. 3, we can observe that the isoelectric point of the analyzed particles is displaced even when we use the same amount of silane on the particles (6 for ethanol and 7 for toluene). As we present in this document, the reaction media induce differences in the morphology of the coating and in the data obtained from the Zeta potential and the Infrared spectra.

## 2 Experimental designd and materials

### 2.1 Experimental design

TiO_2_ nanoparticles were modified superficially by two different silanization processes. The coupling agent: 3-aminopropyl-triethoxy-silane (APTES) was added at a ratio 5:1of (TiO_2_/APTES).

For the incorporation of TiO_2_ in ethanol (Ethanol S-TiO_2_) [1] and in toluene (Toluene S-TiO_2_) [2], we followed the method described in previous reported works.

### 2.2 Materials

We used titanium dioxide (TiO_2_) particles with an average diameter of 350 nm and a crystalline structure of rutile, which were obtained from DuPont (R-104 Dupont, Mexico), 3-aminopropyl-tri-ethoxysilane (Sigma-Aldrich, 97%, Mexico).

## 3 Methods

### 3.1 Transmission electron microscopy (TEM)

The images of the nanoparticles were obtained with a Transmission Electron Microscope JEOL TEM-1010 operating at 100 kV. The nanoparticles were suspended in distilled water and 5 μL of the dilution were placed on copper grids coated with carbon.

### 3.2 Fourier Transform Infrared Spectroscopy (FTIR)

The curves of the spectra were made with the help of a Perkin-Elmer FTIR-ATR spectrometer, Model Spectrum 100. The analyses included range of wave number from 4000 to 1000 cm^−1^ with 60 scans, stacking the nanoparticles for a better reading at 25 °C.

### 3.3 Zeta potential

The Zeta potential tests were performed with the help of a Delsa Nano C particle analyzer A53878 equipment. Solutions of NaOH and HCl at a concentration of 0.001% w/V were used to adjust the pH value using disposable cells equipped with two electrodes in solution for measurements.

The stability of the TiO_2_ particles and the silanized particles (S-TiO_2_) were evaluated in water through their precipitation time, driven by the gravity force at different pH in a range of 2–12, using a light source dual 30 mV and a 658 nm Laser Diode at 25 °C.

## References

[1] López-Zamora L., Martínez-Martínez H.N., González-Calderón J.A. (2018). Improvement of the colloidal stability of titanium dioxide particles in water through silicon based coupling agent. Mater. Chem. Phys..

[2] Kulkarni S.A., Ogale S.B., Vijayamohanan K.P. (2008). Tuning the hydrophobic properties of silica particles by surface silanization using mixed self-assembled monolayers. J. Colloid Interface Sci..

